# Effects of dietary supplementation of vitamin A on the tibia quality of goslings

**DOI:** 10.5713/ab.23.0445

**Published:** 2024-04-25

**Authors:** Xia Xiao, Haiming Yang, Xiaoli Wan, Zhiyue Wang

**Affiliations:** 1 College of Animal Science and Technology, Yangzhou University, Yangzhou, Jiangsu 225009, China; 2 Jiangsu Coastal Area Institute of Agricultural Science, Yancheng, Jiangsu 224002, China; 3 Joint International Research Laboratory of Agriculture and Agri-Product Safety of Ministry of Education of China, Yangzhou University, Yangzhou, Jiangsu 225009, China

**Keywords:** Calcium, Gosling, Phosphorus, Tibia, Vitamin A

## Abstract

**Objective:**

This study was conducted to evaluate the effect of dietary supplementation of vitamin A (VA) on the tibial growth, calcium (Ca) and phosphorus (P) metabolism, VA, and vitamin D (VD) deposition, and associated gene expression in goslings.

**Methods:**

A total of 180 healthy, 1-day-old male goslings were randomly divided into 3 treatment groups (0, 9,000, and 15,000 IU VA/kg), with 6 replicates containing 10 goslings each. They were weighed and sampled on days 14, 28, 42, 56, and 70.

**Results:**

No addition of VA reduced VA content in the serum and liver of goslings, and supplementation of 15,000 IU/kg VA increased VA content from day 14 (p<0.05). The trend of VA concentration in the serum and liver was in line with the relative mRNA expression of retinoic acid receptor β in the jejunal mucosa. In both no addition of VA and supplementation of 15,000 IU/kg VA reduced 25-hydroxycholecalciferol (25-OH-VD_3_) content in the serum and VD content in the liver (p<0.05). From day 28, no addition of VA or supplementation of 15,000 IU/kg VA had a negative effect on tibia length, strength, and Ca, P, and ash content in goslings (p<0.05). Tibia P content was lower in the supplementation of 15,000 IU/kg VA group than in the no addition of VA group (p<0.05). No addition of VA or supplementation of 15,000 IU/kg VA had the most effect on early serum parathyroid hormone (PTH) levels in goslings (p<0.05). The effect of no addition of VA on the bone Gla protein (BGP) content of goslings started from day 14 (p<0.05). The relative mRNA expression of bone Gla-protein (*BGLAP*) and bone morphogenetic protein 4 (*BMP4*) in the liver and jejunal mucosa was decreased by either no addition of VA or supplementation of 15,000 IU/kg VA (p<0.05).

**Conclusion:**

Both no addition of VA and supplementation of 15,000 IU/kg VA affected the mineralization process of the bone, and ultimately reduced tibial quality.

## INTRODUCTION

Bone health has been a longstanding concern in the realm of poultry farming. The most common and harmful illnesses influencing poultry productivity are still metabolic diseases, especially skeletal dysfunction. Locomotor deficiencies arising from such maladies greatly increase the cost of output and carcass processing [1]. It’s interesting to note that while the performance of poultry production has gradually improved due to advancements in genetic breeding, nutrition, and feed science as well as the use of sophisticated technologies, stress resistance has greatly decreased and the frequency of leg illness has grown [2]. The poultry industry has suffered significant losses as a result of the sharp rise in leg disease and the mortality rate [3]. Even if things have improved thanks to genetic breeding and other techniques, poultry leg disease still poses a serious threat to the industry’s further growth. Therefore, researchers continue to focus on studying the etiology, mechanisms, and preventive measures of poultry leg disease from multiple perspectives.

Vitamin A (VA) is a fat-soluble nutrient that promotes the metabolism of substances in the body. VA and retinoic acid derivatives are considered morphological factors in bone formation [4]. Appropriate levels of VA maintain the body’s bone health, and too much or too little VA can have a negative impact on bone growth. According to the definition of the World Health Organization (WHO), a serum VA level of less than 0.7 μmol/L (20 μg/dL) is considered a VA deficiency [5]. However, certain investigators have proposed that a risk of VA insufficiency also exists at VA levels below 1.05 μmol/L [5]. In poultry, recognized symptoms of VA deficiency include stunted growth, ruffled feathers, weakness, dry eyes, impaired egg production, low immunity, and reduced resistance to certain poultry diseases [6]. The marginal deficiency of VA, which is more prevalent in current production, affects growth and development, lower resistance to disease, and other aspects of livestock and poultry significantly but does not manifest clear clinical deficiency signs. In order to boost immunity and achieve greater performance, VA is frequently overdosed during manufacture. The limits of VA overdose are unclear, as different animal species have different levels of tolerance. The National Research Council (NRC, 1994) states that the maximum tolerance for VA in broilers and growing laying hens is 15,000 IU/kg [7]. According to Yan et al [8], dietary VA levels significantly affect the metabolism of phosphorus (P) and calcium (Ca) in broilers. A dietary VA level of 45,000 IU/kg was found to cause a decrease in tibia Ca deposition, an increase in P deposition, and a decrease in tibia mineralization, indicating an excess of VA. The Ca and P metabolisms of broilers were also adversely affected at a dietary VA level of 15,000 IU/kg, indicating a critical excess of VA [8].

The deficiency or excess of VA in the diet can cause a disruption of Ca and P metabolism, impeded bone growth and development, and the appearance of the symptoms of bone deformity [9]. In mice, dry bone weight, ash content, and bone mineralization decreased as the intake of all-trans-retinoic acid increased [10]. According to Conaway et al [11], consuming too much VA is linked to a decrease in bone density in people, which can cause osteoporosis and a higher risk of fractures. It is known that there is a dosage impact between VA and vitamin D (VD), which could be harmful to the body’s bones [12]. Some studies have hypothesized that VA may have an antagonistic effect on VD because it can either compete with VD for absorption at the small intestinal mucosa, causing interference with normal VD absorption and metabolism, or it can partially damage VD before the digested material reaches the site of absorption [13,14]. Moreover, retinoid X receptor (RXR) proteins are necessary for the molecular synthesis of heterodimers by both VA and VD to initiate transcriptional activity [15,16]. If large doses of VA are given in animals in which 9-cis retinoic acid can innervate or utilize many of the available RXR proteins, the effects of VD may be inhibited [13]. It’s possible that VA affects VD absorption and metabolism, which in turn affects bone health and Ca and P metabolism. Further research is necessary to determine the precise mechanism.

The NRC (1994) recommended VA requirement for geese is 1,500 IU/kg [7]. However, it has been shown that the addition of 9,000 IU/kg VA to the diet of 0 to 28 d goslings can meet their growth and development requirements. Additionally, no addition of VA or the addition of 15,000 IU/kg VA is harmful to the growth and development of goslings, and there is an antagonistic relationship between VA and VD [17]. Based on the growth performance and tibia characteristics of goslings, it is recommended that the best dietary VA supplementation for 0 to 28 d goslings should be 9,000 IU/kg [17]. Studies on the effects of VA deficiency and excess on bone metabolism have mainly focused on humans and rats, with fewer studies and inconsistent results in poultry. Thus, this study examined the effects of dietary supplementation of VA in the diets on VA and VD deposition, tibia growth, Ca and P metabolism, and related gene expression in the goslings at different ages to investigate the effects of VA on the tibia quality in poultry and the regulatory pathways. The outcomes of this study will serve as a reference for the prudent application of VA in the production of geese, as well as the experimental and theoretical foundation for the early prevention and treatment of animal legs through dietary management.

## MATERIALS AND METHODS

### Ethics statement

Under Approval No. SYXK [Su] IACUC 2016-0020, all experimental procedures involving animal manipulation were reviewed and approved by the Institutional Animal Care and Use Committee (IACUC) of the Yangzhou University Animal Experiments Ethics Committee (Yangzhou, China).

### Experimental design and diets

The experiment was conducted at Gaoyou Modern Agricultural Farm in Yangzhou, China. A total of 180 healthy male one-day-old Jiangnan White goslings with similar body weights (BWs) (101.3±5.7 g) were produced by the same flock of geese. The goslings were randomized to 3 dietary treatments (6 replicates×10 goslings), and the test period lasted 70 days.

The corn-soybean meal basal diets were formulated to meet the nutritional requirements of goslings according to the NRC (1994) recommendations [7] and prior research results [18,19], except for VA (Table 1). The experimental diets were formulated using the corn-soybean meal basal diet supplemented with 0, 9,000, and 15,000 IU/kg VA. Vitamin A was added as retinyl acetate (1×10^6^ IU/g), purchased from Diesman Vitamin Co., Ltd. (Shanghai, China). Different contents of VA were prepared into premix and then mixed with other feed materials. The concentrations of VA in the various diets were analyzed and found to be 1,225, 10,348, and 16,434 IU/kg during the period of 1 to 28 days, respectively. Similarly, over the period of 29 to 70 days, the concentrations were 1,205, 10,325, and 16,418 IU/kg, respectively. These measurements were conducted using the method of Liang et al [20].

All goslings were reared in plastic wire-floor pens (1.5 m ×1.8 m) equipped with nipple drinkers and a feed trough. Birds had *ad libitum* access to feed and water and were raised under experimental conditions to minimize stress during the rearing period. The room temperature was 26°C ±3°C. The goslings were subjected to 23 h of light exposure per day from 1 to 14 days and 18 h per day from 15 to 28 days. The birds were subjected to natural daylight for a duration of 29 to 70 days. Specific feeding management steps are described by Xiao et al [21].

### Blood and tissue sampling

Goslings were weighed on days 14, 28, 42, 56, and 70 after a 6-h period of fasting. The feed intake was recorded for each replicate to calculate the average daily gain (ADG) and average daily feed intake (ADFI). The feed/gain ratio (F/G) was calculated as the ratio between ADFI and ADG in each replicate. Then, 1 gosling with average BW was selected from each replicate. Vacuum tubes and stainless-steel blood collection needles were used to collect 2 mL of blood from the wing vein. The blood was allowed to clot at 37°C for 2 h. The serum was separated by centrifugation at 2,000×g for 10 min using a Cence DL-5M low-speed frozen centrifuge (Hunan Xiangyi Laboratory Instrument Development Co., Ltd., Changsha, China). After blood collection, the goslings were sacrificed by cervical dislocation and exsanguinated. About 500 mg of liver tissue was promptly extracted and transferred to cryopreservation tubes. The tubes were snap-frozen with liquid nitrogen and then stored in a refrigerator at −80°C for mRNA extraction and VA and VD content analysis.

About 10 cm of the middle part of the jejunum was cut off, and the chyme was drained and washed with normal saline. The inner surface mucosa of the intestinal tract was gently scraped with sterilized slides, put into RNase-free tubes, snap-frozen in liquid nitrogen, and then transferred to a −80°C refrigerator for storage. In addition, the left tibia of the gosling was separated, and the attached tissue was removed and stored in a plastic bag at −20°C to determine tibia strength and mineral element content.

### Serum sample analysis

An enzyme-linked immunosorbent assay was used to determine the values of VA and 25-hydroxycholecalciferol (25-OH-VD_3_) in gosling serum. The kits were purchased from Jiangsu Kete Biotechnology Co., Ltd. (Yancheng, China) and Nanjing Jiancheng Bioengineering Institute (Nanjing, China). The liver concentrations of VA and VD were analyzed with high-performance liquid chromatography (HPLC), following the methodology described by Liang et al [20]. For parathyroid hormone (PTH) and bone Gla protein (BGP) in serum, ELISA kits purchased from Beijing North Biotechnology Research Institute Co., Ltd. (Beijing, China) were used in strict accordance with their operating manual. Serum concentrations of Ca and P were measured using a Beckman LX-20 automatic biochemical analyzer (Beckman Coulter, Inc., Fullerton, CA, USA). Serum Ca and P were assayed by the ion electrode method.

### Bone sample analysis

The tibia length was measured using a vernier caliper (500-703-20 CD-P20S; Mitutoyo Precision Instruments Co., Ltd., Japan) as the distance from the proximal section to the position between the third and fourth toes. The tibia circumference was calculated by wrapping cotton thread around the middle of the tibia for 1 turn and measuring the length of the thread with a straightedge. The tibiae were cleaned of surrounding muscles and soft tissues and measured the strength by a dual column universal testing system (In-stron3367; Instron Corporation, Norwood, MA, USA). The determination procedures were performed according to the protocol outlined by Guo et al [22]. The blade perpendicularly hit the tibia at its midpoint at a test speed of 5 mm/s for 30 mm. The tibia was positioned on the bracket at the same bending degree, and the upper pressure was loaded at a uniform speed until the tibia was broken. The bending load (Newton, N) at the moment of fracture was recorded. Bone fragments from breaking strength determination were oven-dried at 65°C for 24 h. They were then defatted with ether for 7 d. After this, the bone fragments were again oven-dried at 65°C for 24 h to determine dry defatted bone weight. The dry tibiae were crushed, ashed on an electric stove, and burned in a muffle furnace at 550°C for 12 h (SX2-4-10; Shanghai Gengfa Pharmaceutical Equipment Co., Ltd., Shanghai, China). Ash content was calculated as a percentage of the dry fat-free tibia weight. The ash was dissolved with nitric and perchloric acids. According to the procedures of AOAC (1995), the Ca content was determined by titration with ethylene diamine tetraacetie acid (EDTA), and the P was determined via the molybdenum yellow colorimetry method [23]. The ash, Ca, and P contents were expressed based on tibial wet weight.

### RNA extraction, reverse transcription, and real-time quantitative polymerase chain reaction

Total RNA was extracted from liver and jejunum mucosa samples using the Trizol reagent from Tiangen Biochemical Technology Co., Ltd. (Beijing, China). The concentration and purity of the total RNA were measured using a NanoDrop ND-1000 spectrophotometer (Thermo Fisher Scientific, Waltham, MA, USA) based on the absorbance at 260/280 nm. Following concentration determination, 1.0 μg of total RNA was used to create cDNA utilizing reverse transcription with a reagent kit. The cDNA was diluted, and relative quantitative analysis was performed by 7500 real-time PCR instruments (Applied Biosystems, Foster City, CA, USA). All kits were purchased from Yeasen Biotechnology (Shanghai, China) Co., Ltd. The reaction system consisted of 20 μL, with components including Hieff qPCR SYBR Green Master Mix (Low Rox) 10 μL, forward and reverse primers 0.4 μL each, cDNA template 2 μL, and enzyme-free water 7.2 μL, using β-actin as the reference gene. The reaction conditions include pre-denaturation at 95°C for 5 min, denaturation at 95°C for 10 s, and annealing at 60°C for 30 s, repeated for 40 cycles. After the reaction, the relative expression was calculated by the 2^−ΔΔCT^ method. The primer sequences were constructed based on the mRNA of retinoic acid receptor β (*RARB*), bone Gla-protein (*BGLAP*), bone morphogenetic protein 4 (*BMP4*), and *β-actin* from geese (*Anser Cygnoides domesticus*) available in GenBank. All the primers were synthesized by Beijing Qingke Biotechnology Co., Ltd. (Beijing, China). Primer information is presented in Table 2.

### Statistical analysis

All obtained data were analyzed using one-way one way analysis in SPSS 26.0 (SPSS, Inc., Chicago, IL, USA). Each replicate served as an experimental unit for all statistical analyses. The results of the data analysis are expressed as the mean value and the pooled standard error of the mean. Differences among the treatment means were determined at p<0.05 by Tukey’s multiple range test. All the graphs were made using GraphPad Prism 8.0.

## RESULTS

### Growth performance

From day 28, no addition of VA or supplementation of 15,000 IU/kg VA decreased the BW of goslings (p<0.05). There were negative effects of no addition of VA on the BW throughout the experiment, but supplementation of 15,000 IU/kg VA had no significant effect on the BW on days 56 and 70 (p>0.05). No addition of VA decreased the ADG of goslings from 15 to 42 days and increased the F/G of goslings from 15 to 28 days (p<0.05). Supplementation of 15,000 IU/kg VA had no significant effect on ADG and F/G (p>0.05). Xiao et al [21] presented comprehensive data.

### Deposition of vitamin A and vitamin D in the liver and serum

The effects of dietary supplementation of VA in gosling diets on VA and VD contents in the liver and serum at different ages are presented in Table 3. It can be seen from the table that dietary VA content had effects on VA and VD contents in the liver and serum (p<0.05). At each time point, the VA content in the serum and liver of the no addition of VA group was lower than that of the supplementation of 9,000 IU/kg VA group, and the VA content in the supplementation of 15,000 IU/kg VA group was higher (p<0.05). At the same time, 25-OH-VD_3_ in the serum and VD in the liver of the no addition of VA and supplementation of 15,000 IU/kg VA groups were lower than those in the supplementation of 9,000 IU/kg VA group (p<0.05). The serum levels of VA in the supplementation of 9,000 IU/kg VA and 15,000 IU/kg VA groups first increased, then dropped over time, reaching their peak on day 42. The liver VA content in the supplementation of 9,000 IU/kg VA group gradually grew and reached a steady level, but that in the supplementation of 15,000 IU/kg VA group consistently increased. In the no addition of VA group, both serum and liver VA contents decreased gradually. Specifically, compared to the supplementation of 9,000 IU/kg VA group, the serum 25-OH-VD_3_ content in the no addition of VA group decreased by 19.8% on day 14 (p<0.05), the serum VA and liver VD contents decreased by 85.8% (p<0.05) and 24.0% (p<0.05) on day 42, respectively, and liver VA content decreased by 99.7% on day 70 (p<0.05). The serum and liver VA content in the supplementation of 15,000 IU/kg VA group increased by 16.1% (p<0.05) and 621% (p<0.05) on day 14, the serum 25-OH-VD_3_ content decreased by 19.3% on day 14 (p<0.05), and the liver VD content decreased by 16.0% on day 56 (p<0.05). These were the moments when the indicator content had the highest increases or decreases.

### Tibia index and related indexes of calcium and phosphorus

The effects of dietary supplementation of VA in gosling diets on the tibia index at different ages are shown in Table 4. It can be seen from the table that dietary VA content had effects on tibia length and strength of goslings (p<0.05). All tibia indices rose over time. The effects of no addition of VA on the tibia length and strength of goslings began from day 42 and day 28 to the end of the experiment, respectively (p<0.05). The effects of supplementation of 15,000 IU/kg VA on the tibia length and strength in goslings began from day 28 to the completion of the trial (p<0.05). No addition of VA or supplementation of 15,000 IU/kg VA affected tibia circumference only on day 70 (p = 0.037). Among them, compared to the supplementation of 9,000 IU/kg VA group, the tibia length (p<0.05), circumference (p<0.05), and strength (p< 0.05) in the no addition of VA group decreased by 1.50 cm, 0.39 cm, and 117 N on day 70. These in the supplementation of 15,000 IU/kg VA group resulted in a decrease of 1.10 cm (p<0.05), 0.49 cm (p<0.05), and 111 N (p<0.05) on day 70.

Table 5 displays the effects of dietary supplementation of VA on Ca and P-related indexes at different ages. Dietary VA supplemental levels had no impact on serum Ca and P contents (p>0.05). The serum Ca and P content initially rose and subsequently declined over time, peaking on day 42. The contents of Ca, P, and ash in the tibia increased on day 42 and then remained stable. The tibia Ca, P, and ash contents of goslings were influenced by the dietary VA supplementation (p<0.05). Calcium and P contents in the tibia of the no addition of VA and supplementation of 15,000 IU/kg VA groups decreased from day 28 (p<0.05). Additionally, the ash content of the tibia reduced from day 42 (p<0.05). Among them, compared to the supplementation of 9,000 IU/kg VA group, the Ca content of the tibia in the no addition of VA group decreased by 16.4% on day 28 (p<0.05), the P content of the tibia decreased by 21.3% on day 42 (p<0.05), and the content of ash in the tibia decreased by 15.1% on day 70 (p< 0.05). The Ca content of the tibia in the supplementation of 15,000 IU/kg VA group decreased by 14.0% on day 28 (p< 0.05), the P content of the tibia decreased by 27.8% on day 56 (p<0.05), and the content of ash in the tibia decreased by 17.2% on day 42 (p<0.05). In addition, the content of tibial P in the supplementation of 15,000 IU/kg VA group was lower than that in the no addition of VA group (p<0.05).

### Content of parathyroid hormone and bone Gla protein in the serum

Table 6 summarizes the effects of dietary supplementation of VA in gosling diets on PTH and BGP contents at different ages. Dietary VA content influenced PTH and BGP contents in goslings (p<0.05). No addition of VA or supplementation of 15,000 IU/kg VA had an impact on the serum PTH content of goslings in the early stages (p<0.05). The impact diminished over time until it was no longer detectable in the later phases. There was a significant decrease in BGP content in goslings from day 28 to the completion of the trial when no VA was added (p<0.05). Supplementation of 15,000 IU/kg VA did not affect BGP content in goslings (p>0.05). Additionally, compared to the supplementation of 9,000 IU/kg VA group, the content of PTH in the no addition of VA group decreased 46.6% by day 14 (p<0.05), the PTH content increased 24.5% by day 42 (p<0.05), and the content of BGP decreased 26.0% by day 56 (p<0.05). The content of PTH in the supplementation of 15,000 IU/kg VA group decreased 39.2% by day 14 (p<0.05).

### Gene expression

**Figure 1:**
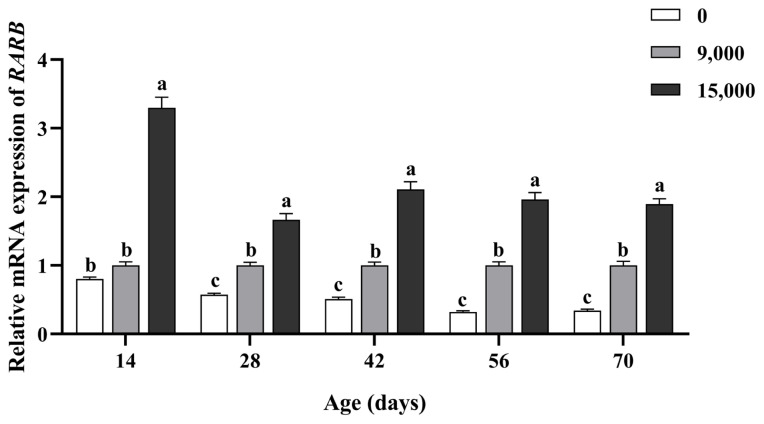
Effects of dietary supplementation of vitamin A on the relative mRNA expression levels of *RARB* in jejunal mucosa of goslings at different ages. Data are represented with the means±standard error of the mean, n = 6. *RARB*, retinoic acid receptor β. ^a–c^ Different letters represent a significant difference (p<0.05).

As shown in Figure 1, compared to the supplementation of 9,000 IU/kg VA group, no addition of VA down-regulated the relative mRNA expression of *RARB* in the jejunal mucosa of goslings from day 28 (p<0.05). Conversely, supplementation of 15,000 IU/kg VA up-regulated the relative mRNA expression of *RARB* in the jejunal mucosa from day 14 (p<0.05).

**Figure 2:**
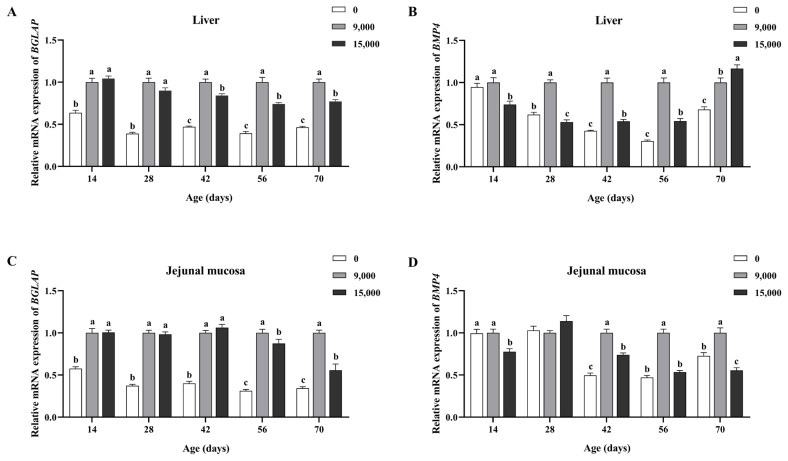
Effects of dietary supplementation of vitamin A on the relative mRNA expression levels in the goslings at different ages. (A) and (B) respectively represent the mRNA expression of *BGLAP* and *BMP4* in liver; (C) and (D) respectively represent the mRNA expression of *BGLAP* and *BMP4* in jejunum mucosa. Data are represented with the means±standard error of the mean, n = 6. *BGLAP*, bone gla protein; *BMP4*, bone morphogenetic protein 4. ^a–c^ Different letters represent a significant difference (p<0.05).

As shown in Figures 2A and 2B, compared to the supplementation of 9,000 IU/kg VA group, no addition of VA reduced the relative mRNA expression of *BGLAP* in the liver of goslings from day 14 and decreased the relative mRNA expression of *BMP4* in the liver from day 28 (p<0.05). Supplementation of 15,000 IU/kg VA reduced the relative mRNA expression of *BGLAP* in the liver from day 42, lowered the relative mRNA expression of *BMP4* from day 14, and increased the relative mRNA expression of *BMP4* on day 70 (p<0.05).

As shown in Figures 2C and 2D, compared to the supplementation of 9,000 IU/kg VA group, no addition of VA decreased the relative mRNA expression of *BGLAP* in the jejunal mucosa of goslings from day 14. It reduced the relative mRNA expression of *BMP4* in the jejunum mucosa from day 42 (p<0.05). Supplementation of 15,000 IU/kg VA decreased the relative mRNA expression of *BGLAP* in the jejunum mucosa from day 56 and decreased the relative mRNA expression of *BMP4* from day 14 (p<0.05).

## DISCUSSION

Vitamin A is crucial for maintaining normal bone growth and metabolism. Inadequate or excessive amounts might harm bone growth and development. The amount of VA supplied to the diet is closely linked to the VA levels in the body. Dietary VA content significantly impacts the *in vivo* metabolism of VA [5,17]. More than 50% to 80% of the body’s VA reserves are in the liver [24]. There was a positive correlation between liver VA concentration and dietary VA concentration. Serum and liver VA levels are commonly used as markers of VA nutritional status in animals. This test showed that no addition of VA decreased VA levels in the serum and liver of goslings, whereas supplementation of 15,000 IU/kg VA increased VA deposition in tissues. It was further verified that the amount of VA added to the diet affected VA deposition in animal tissues. VA was accumulated in the body over time and eventually reached a steady level at a specific age. However, the group of goslings without added VA was unable to obtain sufficient VA from the feed. Consequently, they had to rely on the VA stored in their liver, leading to a gradual depletion of VA in their tissues. Most of the VA ingested by the goslings in the supplementation of 15,000 IU/kg VA group was deposited in the body, resulting in a gradual increase VA accumulation in the tissues over time. Alterations in growth performance may be associated with variations in VA concentration [21].

Rohde and DeLuca [10] found a weak antagonism between VA and VD. Serum 25-(OH)-VD_3_ concentration is a crucial indicator of VD status in the body [25]. Simultaneously, 25-(OH)-VD_3_ has a role in the absorption and utilization of Ca and P and is necessary for mineralizing animal bones [25]. This experiment concluded that the amount of VA added to the diet affected the serum 25-(OH)-VD_3_ and liver VD content. The serum 25-(OH)-VD_3_ content and liver VD content of the no addition of VA and supplementation of 15,000 IU/kg VA groups in goslings were lower than those of the supplementation of 9,000 IU/kg VA group. It could be connected to the interaction between VA and VD [10,12]. Excessive VA may harm VD molecules or compete with VD for absorption and transport sites in the mucosa of the small intestine before the digested material reaches the site of absorption, resulting in lower VD levels [13,14]. The effect can lead to disturbances in Ca and P metabolism in animals.

Bone growth involves longitudinal development and transverse growth. Longitudinal growth increases bone length, while transverse growth increases bone cross-section and diameter. Bone strength is a key factor in evaluating the bone growth of geese. Vitamin A deficiency can lead to increased organic deposition in animal bones. Excessive VA can cause accelerated bone resorption, bone fragility, and spontaneous bone fractures in animals [11]. Guo et al [22] demonstrated that a shortage in VA reduced tibia diameter in laying hens, and excessive VA did not enhance tibia quality. The study found that neither no addition of VA nor supplementation of 15,000 IU/kg VA had a negative effect on the tibial attributes in goslings. It aligns with the results mentioned before. No addition of VA or supplementation of 15,000 IU/kg VA may be arresting growth by inhibiting the proliferation and hypertrophy of growth plate chondrocytes and reducing matrix synthesis via retinoic acid receptors (RARs) [26]. Osteogenesis within the cartilage was retarded, and the differentiation of osteoblasts was hindered, which ultimately affected the longitudinal growth of the bone [27]. Research has demonstrated that an increase in body size is first indicated by growth in tibia circumference, followed by tibia length [28]. In this trial, the negative effect of no addition of VA or supplementation of 15,000 IU/kg VA on tibia length was earlier in the goslings, probably because the VA ingested by the goslings in the early stages preferentially met the tibia circumference growth.

The contents of Ca and P primarily indicate the bone metabolism of animals, with tibial ash serving as the most direct index reflecting bone quality. Minerals accumulating on the periosteum surface enhanced tibial strength as the individual aged [29]. High levels of VA affect the bone development of poultry, leading to lower bone ash and Ca content, less bone mineralization, and an increased risk of leg disorders [30]. More studies have focused on Ca and P metabolism in mice or broilers in relation to VA deficiency or excess, with excess being the more prevalent issue. Stevens et al [31] showed that dietary supplementation with high levels of VA reduced bone weight and bone ash content in broilers. Mir et al [32] demonstrated that insufficient or excessive VA supplementation in the diet can reduce the bone ash and Ca content in broilers. Several investigations have found that the effect of dietary VA levels in the diet on serum Ca and P levels is negligible. The impact on tibial Ca, P, and ash levels was more noticeable, suggesting that tibial Ca and P metabolism is more sensitive to VA levels [17]. The findings of this test were similar to the above tests. Neither no addition of VA nor supplementation of 15,000 IU/kg VA affected serum Ca and P levels in geese. However, both no addition of VA and supplementation of 15,000 IU/kg VA altered tibial Ca, P, and ash levels in goslings.

Various factors, such as PTH and BGP, can regulate the metabolic utilization and homeostasis of Ca and P in the serum and bones of animals. Parathyroid hormone is essential for maintaining normal Ca, phosphate homeostasis, and bone strength. Plasma Ca ions negatively regulate PTH synthesis and secretion [33]. In this experiment, on days 14 and 28, serum PTH content in the no addition of VA and supplementation of 15,000 IU/kg VA groups was lower than that in the supplementation of 9,000 IU/kg VA group. However, on days 42 and 56, PTH levels increased. Although the serum Ca content had no effect, from the data point of view, it showed a trend opposite to that of PTH content. When VA was deficient or critically excessive, the serum Ca content increased early. It inhibited the synthesis and secretion of PTH [33]. At the same time, the serum Ca content declined at a later stage, stimulating the synthesis and secretion of PTH to ultimately stabilize serum Ca concentration. Therefore, fluctuations in serum PTH levels in response to VA deficiency or excess are an adaptive adjustment of Ca metabolism disorder.

Research on BGP has shown that the BGP concentration in the bloodstream is indicative of osteoblast activity, bone formation rate, and serves as a specific biochemical indicator of bone turnover [34]. Guo et al [27] showed that VA inhibited BGP levels in broiler osteoblast cultures in a secondary dose-dependent manner as VA levels increased. Results indicated that from day 28, serum BGP content in the no addition of VA group was lower than that in the supplementation of 9,000 IU/kg VA group. No addition of VA caused a decrease of BGP content in the serum of goslings, indicating a decrease in osteoblast activity and an inhibition of the bone formation rate in goslings. The negative effects of no addition of VA on bone growth and Ca and P metabolism are associated with reduced osteoblast activity and impaired BGP biosynthesis, which in turn affects bone mineralization processes [27].

Vitamin A is involved in cell growth and differentiation by regulating gene expression through the RAR and RXR pathways [4,35]. Li et al [36] found that the effect of VA intake on the expression of RARs is dose-dependent, with RARβ being crucial for embryonic skeletal development. Previous studies have demonstrated that RARβ is expressed in the developing intestine [37]. This experiment showed that from day 14, no addition of VA led to a decrease in VA content in the liver and serum, as well as a reduction in *RARB* mRNA expression in the jejunum of goslings. In contrast, supplementation of 15,000 IU/kg VA increased VA content in the serum and liver and up-regulated *RARB* mRNA expression in the jejunum. Vitamin A deficiency reduced the number of ligands and inhibited *RAR* expression. Its cascade effect can promote the teratogenic effect of VA deficiency on skeletal development and may even lead to embryonic death [36]. It is possible that no addition of VA or supplementation of 15,000 IU/kg VA could be affecting the bone quality of the goslings by affecting *RARB* mRNA expression and perhaps causing the death of the goslings [21]. In addition, retinoic acid, a metabolic intermediate of VA, has been found to inhibit *BGP* and *BMP* mRNA gene expression as well as bone mineralization [38,39]. Bone morphogenetic protein 4 belongs to the transforming growth factor-β superfamily. The BMP signaling pathway is implicated in tooth development, cell differentiation, and bone formation [40]. The results of this experiment revealed that either no addition of VA or supplementation of 15,000 IU/kg VA down-regulated the relative expression of *BGLAP* and *BMP4* mRNA in tissues. Taken together with the conclusions of Guo et al [27], the adverse effects of no addition of VA or supplementation of 15,000 IU/kg VA on bone quality may be associated with decreased *BGLAP* and *BMP4* gene expression in tissues. Currently, there is a scarcity of study reports in this field, and the precise mechanism of the effect requires additional investigation.

In conclusion, no addition of VA reduced tissue VA and VD deposition in goslings. The mRNA expression of *RARB*, *BGLAP*, and *BMP4* in tissues was down-regulated, and serum BGP levels was lowered. Ultimately, it affected the mineralization process of the tibia. However, supplementation of 15,000 IU/kg VA increased tissue VA deposition and inhibited tissue VD deposition in goslings. It up-regulated *RARB* mRNA expression and down-regulated *BGLAP* and *BMP4* mRNA expression in tissues, leading to a deleterious impact on the tibia Ca and P concentrations. The effect of supplementation of 15,000 IU/kg VA was even more remarkable in tibia P level.

## Figures and Tables

**Table 1:** Composition and nutrient content of the basal diets^1)^

Items	Growth period	

	1 to 28 d	29 to 70 d
Ingredients (%)		
Corn	63.0	60.7
Soybean meal-43%	30.2	24.6
Rice husk	3.20	7.70
Wheat bran	-	3.30
DL-methionine	0.10	0.12
Salt	0.30	0.30
Limestone	1.10	1.02
Calcium hydrogen phosphate	1.10	1.26
Vitamin and trace mineral premix^2)^	1.00	1.00
Calculated nutrient content^3)^ (%)		
Metabolizable energy (MJ/kg)	11.34	11.09
Dry matter	84	84
Ash	3.15	3.94
Crude protein	19.0	16.2
Crude fat	2.87	2.68
Neutral detergent fibre	10.3	10.6
Acid detergent fibre	4.85	4.67
Calcium	0.83	0.87
Total phosphorus	0.56	0.65
Available phosphorus	0.32	0.42
Lysine	0.99	0.82
Methionine	0.42	0.36
Vitamin A (IU/kg)	1,225	1,205

1) No vitamin A added.

2) 1–28 days, provided per kilogram of complete diet (without VA): 3,000 IU vitamin D (rachitasterol), 18 IU vitamin E (D-a-tocopherol), 1.5 mg vitamin K (coagulation vitamin), 0.9 mg vitamin B_1_ (thiamine), 8 mg vitamin B_2_ (riboflavin), 3.2 mg vitamin B_6_ (pyridoxine), 12 μg vitamin B_12_ (cobalamin), 45 mg nicotinic acid, 11 mg pantothenic acid, 0.65 mg folic acid, 50 μg biotin, 60 mg Fe (ferrous sulfate), 10 mg Cu (copper sulfate), 95 mg Mn (manganese sulfate), 90 mg Zn (zinc sulfate), 0.5 mg I (potassium iodide), and 0.2 mg Se (sodium selenite). 29–70 days, provided per kilogram of complete diet (without VA): 3,000 IU vitamin D (rachitasterol), 18 IU vitamin E (D-a-tocopherol), 1.5 mg vitamin K (coagulation vitamin), 0.6 mg vitamin B_1_ (thiamine), 6 mg vitamin B_2_ (riboflavin), 2 mg vitamin B_6_ (pyridoxine), 10 μg vitamin B_12_ (cobalamin), 30 mg nicotinic acid, 9 mg pantothenic acid, 0.5 mg folic acid, 40 μg biotin, 60 mg Fe (ferrous sulfate), 10 mg Cu (copper sulfate), 95 mg Mn (manganese sulfate), 90 mg Zn (zinc sulfate), 0.5 mg I (potassium iodide), and 0.2 mg Se (sodium selenite).

3) Vitamin A is the measured value, and the rest are calculated values.

**Table 2:** Primer sequences of genes

Gene	GenBank ID	Primer sequence (5′ →3′)	Product size (bp)
*BGLAP*	XM_013196523.1	F: GCACTGCTCGTCTTGACTCT	206
		R: CAACGCTCATGCATCTGCTC	
*BMP4*	XM_013193881.1	F: AACCGAATGCTGATGG	224
		R: ACGGCTGACTTGCTG	
*RARB*	XM_013172012.1	F: CCGAAAGAGACGACCCAACA	88
		R: TGCACCTTTTGCACTGATGC	
*β-actin*	XM_013174886.1	F: GCACCCAGCACGATGAAAAT	150
		R: GACAATGGAGGGTCCGGATT	

*BGLAP*, bone Gla-protein; *BMP4*, bone morphogenetic protein 4; *RARB*, retinoic acid receptor β.

**Table 3:** Effects of dietary supplementation of vitamin A on the serum, liver vitamin A and vitamin D deposition in goslings^1)^

Items	Vitamin A supplemented concentration (IU/kg)			SEM	p-value

	0	9,000	15,000		
Serum vitamin A content (ng/mL)					
Day 14	107^c^	112^b^	130^a^	2.45	<0.001
Day 28	121^c^	136^b^	144^a^	2.38	<0.001
Day 42	78.2^c^	550^b^	636^a^	61.0	<0.001
Day 56	79.2^c^	502^b^	623^a^	57.4	<0.001
Day 70	71.8^b^	494^a^	573^a^	55.0	<0.001
Serum 25-OH-VD_3_ content (ng/mL)					
Day 14	51.1^b^	63.7^a^	51.4^b^	2.04	0.007
Day 28	80.0^b^	93.5^a^	85.6^ab^	2.14	0.024
Day 42	91.0^b^	105^a^	94.3^b^	1.97	0.004
Day 56	93.0^b^	105^a^	95.6^b^	1.75	0.003
Day 70	91.6^b^	105^a^	95.7^b^	1.80	0.001
Liver vitamin A content (mg/kg)					
Day 14	2.62^c^	33.0^b^	238^a^	25.6	<0.001
Day 28	1.07^c^	206^b^	411^a^	40.8	<0.001
Day 42	0.960^c^	293^b^	467^a^	47.1	<0.001
Day 56	0.890^c^	317^b^	511^a^	51.4	<0.001
Day 70	0.810^c^	318^b^	543^a^	54.8	<0.001
Liver vitamin D content (mg/kg)					
Day 14	1.15	1.07	1.12	0.024	0.394
Day 28	1.29^c^	1.48^a^	1.39^b^	0.021	<0.001
Day 42	0.897^b^	1.18^a^	1.02^b^	0.038	0.003
Day 56	0.953^b^	1.19^a^	1.00^b^	0.034	0.004
Day 70	0.945^b^	1.23^a^	1.04^b^	0.040	0.006

SEM, standard error of the mean.

1) Each value represents the mean of 6 replicates (n = 6).

a–c Means with different superscripts within the same row indicate a significant difference (p<0.05).

**Table 4:** Effects of dietary supplementation of vitamin A on the tibia index of goslings^1)^

Items	Vitamin A supplemented concentration (IU/kg)			SEM	p-value

	0	9,000	15,000		
Length (cm)					
Day 14	7.73	7.45	7.31	0.102	0.248
Day 28	8.98^ab^	9.51^a^	8.53^b^	0.156	0.026
Day 42	11.2^b^	11.8^a^	11.6^ab^	0.097	0.006
Day 56	11.9^b^	13.0^a^	12.2^b^	0.177	0.010
Day 70	12.0^b^	13.5^a^	12.4^b^	0.232	0.017
Circumference (cm)					
Day 14	3.47	3.43	3.47	0.036	0.920
Day 28	4.40	4.43	4.32	0.037	0.446
Day 42	5.00	5.23	5.13	0.055	0.233
Day 56	5.55	5.42	5.40	0.086	0.761
Day 70	5.58^b^	5.97^a^	5.48^b^	0.085	0.037
Strength (N)					
Day 14	90.6	91.5	88.7	1.52	0.769
Day 28	199^b^	239^a^	185^b^	6.42	<0.001
Day 42	453^b^	560^a^	481^b^	17.6	0.025
Day 56	685^b^	797^a^	725^ab^	17.7	0.019
Day 70	724^b^	841^a^	730^b^	19.0	0.008

SEM, standard error of the mean.

1) Each value represents the mean of 6 6 replicates (n = 6).

a,b Means with different superscripts within the same row indicate a significant difference (p<0.05).

**Table 5:** Effects of dietary supplementation of vitamin A on the content of calcium and phosphorus in serum and tibia of goslings^1)^

Items	Vitamin A supplemented concentration (IU/kg)			SEM	p-value

	0	9,000	15,000		
Serum calcium (mmol/L)					
Day 14	2.28	2.25	2.29	0.030	0.872
Day 28	2.16	2.27	2.16	0.028	0.226
Day 42	3.49	3.63	3.52	0.052	0.533
Day 56	3.46	3.51	3.43	0.058	0.884
Day 70	3.34	3.48	3.37	0.063	0.690
Serum phosphorus (mmol/L)					
Day 14	2.45	2.53	2.51	0.099	0.945
Day 28	1.60	1.62	1.46	0.037	0.141
Day 42	2.53	2.65	2.61	0.058	0.698
Day 56	2.32	2.57	2.33	0.059	0.128
Day 70	2.34	2.61	2.37	0.057	0.098
Tibia calcium (g/kg)					
Day 14	89.1	94.6	89.7	1.78	0.401
Day 28	91.1^b^	109^a^	93.7^b^	3.21	0.030
Day 42	114^b^	123^a^	113^b^	1.87	0.046
Day 56	111^b^	125^a^	116^b^	1.86	0.006
Day 70	114^b^	123^a^	116^b^	1.44	0.012
Tibia phosphorus (g/kg)					
Day 14	37.2	38.9	37.6	0.396	0.186
Day 28	44.3^b^	50.6^a^	38.3^c^	1.54	0.001
Day 42	42.9^b^	54.5^a^	39.6^b^	1.67	<0.001
Day 56	43.2^b^	54.4^a^	39.3^c^	1.62	<0.001
Day 70	42.7^b^	53.4^a^	39.8^c^	1.50	<0.001
Tibia ash (g/100 g)					
Day 14	21.8	22.8	21.0	0.407	0.207
Day 28	22.3	24.7	20.6	0.717	0.052
Day 42	24.2^b^	28.5^a^	23.6^b^	0.575	<0.001
Day 56	25.0^b^	28.7^a^	24.0^b^	0.524	<0.001
Day 70	23.6^b^	27.8^a^	23.3^b^	0.527	<0.001

SEM, standard error of the mean.

1) Each value represents the mean of 6 6 replicates (n = 6).

a–c Means with different superscripts within the same row indicate a significant difference (p<0.05).

**Table 6:** Effects of dietary supplementation of vitamin A on the serum parathyroid hormone and bone Gla protein contents in goslings^1)^

Items	Vitamin A supplemented concentration (IU/kg)			SEM	p-value

	0	9,000	15,000		
PTH (pg/mL)					
Day 14	819^b^	1,535^a^	933^b^	83.6	<0.001
Day 28	984^b^	1,435^a^	1,094^b^	58.9	0.001
Day 42	1,609^a^	1,292^b^	1,328^b^	48.8	0.017
Day 56	1,509^a^	1,256^b^	1,346^ab^	43.4	0.050
Day 70	1,438	1,293	1,300	39.4	0.067
BGP (ng/mL)					
Day 14	21.4^a^	18.5^b^	18.4^b^	0.568	0.039
Day 28	14.7^b^	18.3^a^	17.6^a^	0.474	0.001
Day 42	12.6^b^	16.5^a^	16.1^a^	0.533	0.001
Day 56	11.1^b^	15.0^a^	15.0^a^	0.544	<0.001
Day 70	9.72^b^	11.7^a^	10.2^ab^	0.355	0.045

SEM, standard error of the mean; PTH, parathyroid hormone; BGP, bone Gla protein.

1) Each value represents the mean of 6 6 replicates (n = 6).

a,b Means with different superscripts within the same row indicate a significant difference (p<0.05).
